# A Practical Torque Estimation Method for Interior Permanent Magnet Synchronous Machine in Electric Vehicles

**DOI:** 10.1371/journal.pone.0130923

**Published:** 2015-06-26

**Authors:** Zhihong Wu, Ke Lu, Yuan Zhu

**Affiliations:** 1 School of Automotive Engineering, Tongji University, Shanghai, China; 2 Sino-German School for Postgraduate Studies, Tongji University, Shanghai, China; University of California Berkeley, UNITED STATES

## Abstract

The torque output accuracy of the IPMSM in electric vehicles using a state of the art MTPA strategy highly depends on the accuracy of machine parameters, thus, a torque estimation method is necessary for the safety of the vehicle. In this paper, a torque estimation method based on flux estimator with a modified low pass filter is presented. Moreover, by taking into account the non-ideal characteristic of the inverter, the torque estimation accuracy is improved significantly. The effectiveness of the proposed method is demonstrated through MATLAB/Simulink simulation and experiment.

## Introduction

Vehicles powered by a conventional internal combustion engine are extremely less efficient and produce numerous greenhouse gas. As a consequence, great efforts have been made by companies and research institutes to increase the efficiency of energy usage and reduce greenhouse gas emissions of vehicles. The electrification of the vehicle powertrain system is considered as one of the most promising solutions [1–3]. Electric vehicles, including battery electric vehicles (BEVs), hybrid electric vehicles (HEVs), fuel cell electric vehicles (FCEVs), have become more and more popular in the last 15 years.

And in the powertrain system of electric vehicles, two types of eclectic machines are widely used, i.e. induction machines and Permanent Magnet Synchronous Machines (PMSM). Compared with induction machines, PMSM can achieve a very good overall power density and energy efficiency with a relatively small, compact design. And over the last ten years, PMSM are more preferred in HEVs and BEVs than induction machines [4]. Among different types of PMSM, Interior Permanent Magnet Synchronous Machines (IPMSM) have higher power density than Surface Mounted Permanent Magnet Synchronous Machines (SPMSM) because IPMSM can produce extra reluctance torque, and are more suitable for electric vehicles.

In the IPMSM drive in electric vehicles, Maximum Torque per Ampere (MTPA) control strategy is often used to reduce the copper loss [5]. But the major problem about the conventional MTPA method is that the torque output accuracy is influenced by variations of the stator inductance and the flux of the rotor. MTPA compensation methods considering the inductance variation presented in [6–8] and compensation methods considering the variation of the rotor flux presented in [9–11] are investigated to improve the torque output accuracy, but these methods are hard to implement in mass production, because variation patterns of these parameters have to be identified offline for each motor.

Consequently, a redundant torque estimation method is necessary out of safety consideration, to ensure that the torque output is in a reasonable range. Many torque estimation methods have been developed, for example, the torque estimation method based on the Sliding Mode Observer (SMO) presented in [12], the torque estimation method based on the adaptive observer presented in [13], and the torque estimation method based on the flux observer presented in [14,15]. Compared with other methods, the torque estimation method based on the flux observer has the best robustness to parameter variations. Besides, this method can be carried out in the stationary reference frame, so the estimation accuracy doesn’t rely on the accuracy of the rotor position information. The disadvantage of the torque estimation method based on the flux observer is that a pure integrator is often used in this method and it will lead to an accumulating error. In [16], the estimation error caused by the pure integrator is eliminated by using a Modified Low Pass Filter (MPLF).

However, in these methods the influence of the inverter are not considered. In this paper, a practical torque estimation method for IPMSM is presented. It is based on the stator flux observer with MLPF, and the compensation method for MLPF is derived. The major contribution of this paper is that, by taking into consideration the dead-time effect and the non-ideal characteristic of the inverter, reference voltages of the inverter are carefully corrected and then used in the stator flux observer, so a good estimation accuracy can be achieved without additional voltage transducers. Simulation and experimental results show the performance of the proposed method.

The remainder of this paper is organized as follows: in section ‘Materials and Methods’, the conventional torque estimation methods based on the flux observer with a pure integrator and with MLPF are introduced, then the voltage correction method and the proposed torque estimation method are presented. In section ‘Results and Discussion’, the simulation and experimental tests are carried out and the results are investigated. Conclusions are drawn in the last section.

## Materials and Methods

### Conventional Torque Estimation Based on the Stator Flux Observer

#### Torque Estimation Method with the Pure Integrator

The voltage equation of IPMSM in the stationary reference frame is given as:

$$\left\{ \begin{array}{l} V_{\alpha }=R_{s}i_{\alpha }+\frac{d\psi_{\alpha }}{dt}\\ V_{\beta }=R_{s}i_{\beta }+\frac{d\psi_{\beta }}{dt} \end{array}\right.$$ (1)

where *V*_*α*_, *V*_*β*_, *i*_*α*_, *i*_*β*_ and *ψ*_*α*_, *ψ*_*β*_ are stator voltages, stator currents and stator flux respectively in the stationary reference frame, and *R*_*s*_ is the stator resistance.

**Fig 1:**
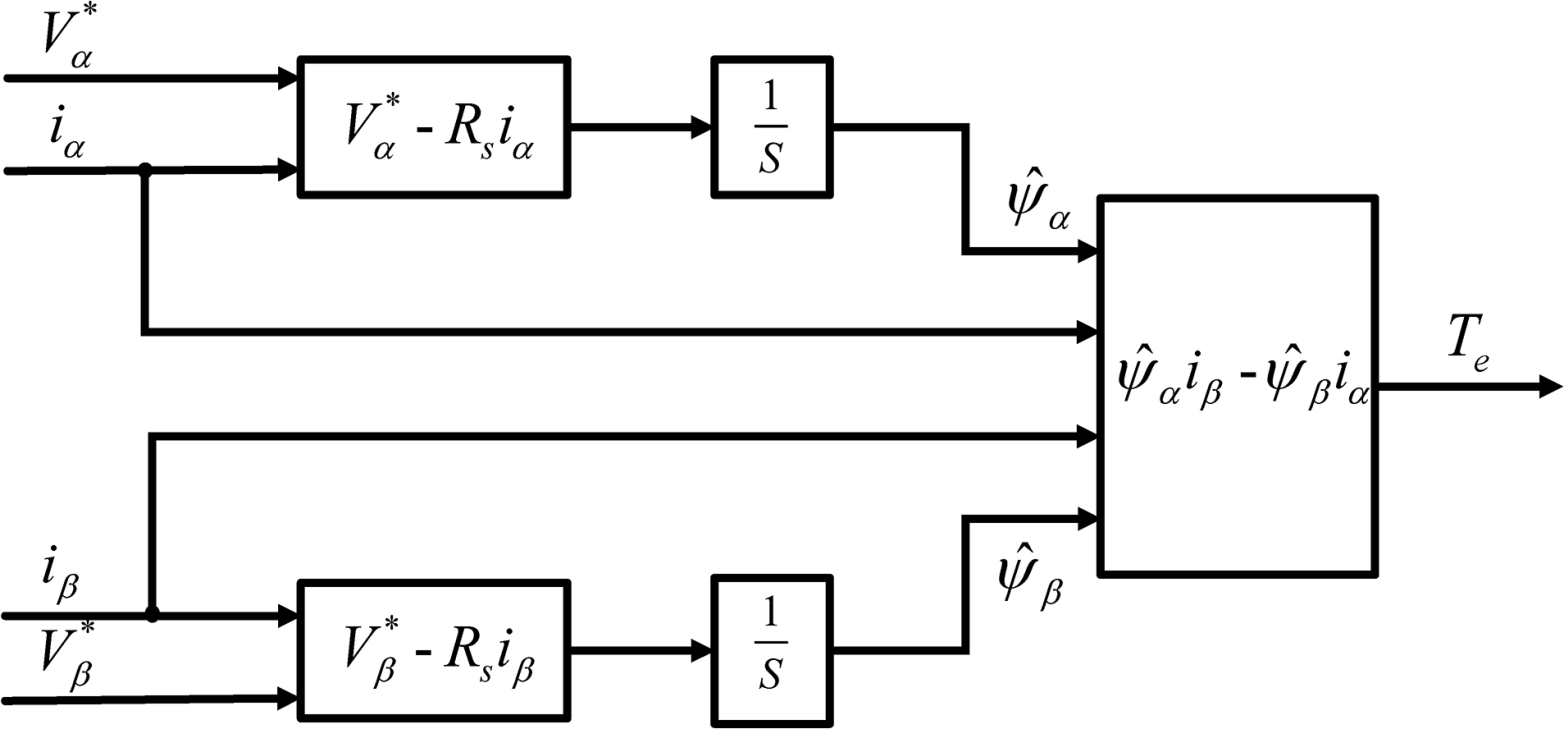
Torque Estimation Method with Pure Integrator.

Instead of using actual stator voltages, reference voltages can be used to eliminate costs of voltage transducers, so stator flux linkages of the IPMSM can be estimated as:

$$\left\{ \begin{array}{l} {\hat{\psi }}_{\alpha }=\int \left(V_{\alpha }^{*}-R_{s}i_{\alpha }\right)dt\\ {\hat{\psi }}_{\beta }=\int \left(V_{\beta }^{*}-R_{s}i_{\beta }\right)dt \end{array}\right.$$ (2)

where $V_{\alpha }^{*}$, $V_{\beta }^{*}$ are reference voltages. And the estimated electromagnetic torque ${\hat{T}}_{e}$ can be calculated from the estimated flux linkages as:

$${\hat{T}}_{e}=\frac{3}{2}p({\hat{\psi }}_{\alpha }i_{\beta }-{\hat{\psi }}_{\beta }i_{\alpha })$$ (3)

where *p* is the pole pairs number of IPMSM. The schematic diagram of the described torque estimation method is shown in Fig 1.

#### Torque Estimation Method with the Modified Low Pass Filter

In practical applications, the pure integrator is easily influenced by the DC drift and the initial value error. In order to solve this problem, the pure integrator can be replaced by a Modified Low Pass Filter (MLPF), and the transfer function of MLPF is given as:

$$G(s)=\frac{1}{s+\omega_{c}}$$ (4)

where *ω*_*c*_ is the cut off frequency of MLPF.

The phase lag *ϕ* and the gain *M* of MLPF are given respectively as:

$$\left\{ \begin{array}{l} \phi ={\text{tan}}^{-1}(\frac{\omega_{e}}{\omega_{c}})\\ M=\frac{1}{\sqrt{\omega_{e}^{2}+\omega_{c}^{2}}} \end{array}\right.$$ (5)

where *ω*_*e*_ is the operation frequency of IPMSM.

The phase lag of the pure integrator is 90° and the gain is 1 / |*ω*_e_|, so there are phase error and gain error between MLPF and the pure integrator, and the errors increase when the operating frequency gets close to the cut off frequency of MLPF. For better estimation accuracy, the outputs of MLPF need to be compensated. Actually, MLPF can be expressed as:

$$G(s)=\frac{1}{s}\cdot \frac{s}{s+\omega_{c}}$$ (6)

Eq 6 means MLPF can be seemed as a pure integrator cascaded with a high pass filter. So the estimated flux linkages ${\hat{\psi }}_{\alpha }^{\prime }(s)$, ${\hat{\psi }}_{\beta }^{\prime }(s)$ using MLPF can be expressed as:

$$\left\{ \begin{array}{l} {\hat{\psi }}_{\alpha }^{\prime }(s)={\hat{\psi }}_{\alpha }(s)\cdot \frac{s}{s+\omega_{c}}\\ {\hat{\psi }}_{\beta }^{\prime }(s)={\hat{\psi }}_{\beta }(s)\cdot \frac{s}{s+\omega_{c}} \end{array}\right.$$ (7)

And Eq 7 can be rewritten as:

$$\left\{ \begin{array}{l} {\hat{\psi }}_{\alpha }(s)={\hat{\psi }}_{\alpha }^{\prime }(s)\cdot \frac{s+\omega_{c}}{s}\\ {\hat{\psi }}_{\beta }(s)={\hat{\psi }}_{\beta }^{\prime }(s)\cdot \frac{s+\omega_{c}}{s} \end{array}\right.$$ (8)

Then the compensation method can be derived as:

$$\left\{ \begin{array}{l} {\hat{\psi }}_{\alpha }(s)={\hat{\psi }}_{\alpha }^{\prime }(s)+{\hat{\psi }}_{\alpha }^{\prime }\cdot \frac{\omega_{c}}{s}\\ {\hat{\psi }}_{\beta }(s)={\hat{\psi }}_{\beta }^{\prime }(s)+{\hat{\psi }}_{\beta }^{\prime }\cdot \frac{\omega_{c}}{s} \end{array}\right.$$ (9)

Considering the demerit of the pure integrator, and assuming the magnitude and the frequency of the stator flux linkages are constant, the compensation method in time domain can be simplified as:

$$\left\{ \begin{array}{l} {\hat{\psi }}_{\alpha }(t)={\hat{\psi }}_{\alpha }^{\prime }(t)+{\hat{\psi }}_{\beta }^{\prime }(t)\cdot \frac{\omega_{c}}{\omega_{e}}\\ {\hat{\psi }}_{\beta }(t)={\hat{\psi }}_{\beta }^{\prime }(t)-{\hat{\psi }}_{\alpha }^{\prime }(t)\cdot \frac{\omega_{c}}{\omega_{e}} \end{array}\right.$$ (10)

**Fig 2:**
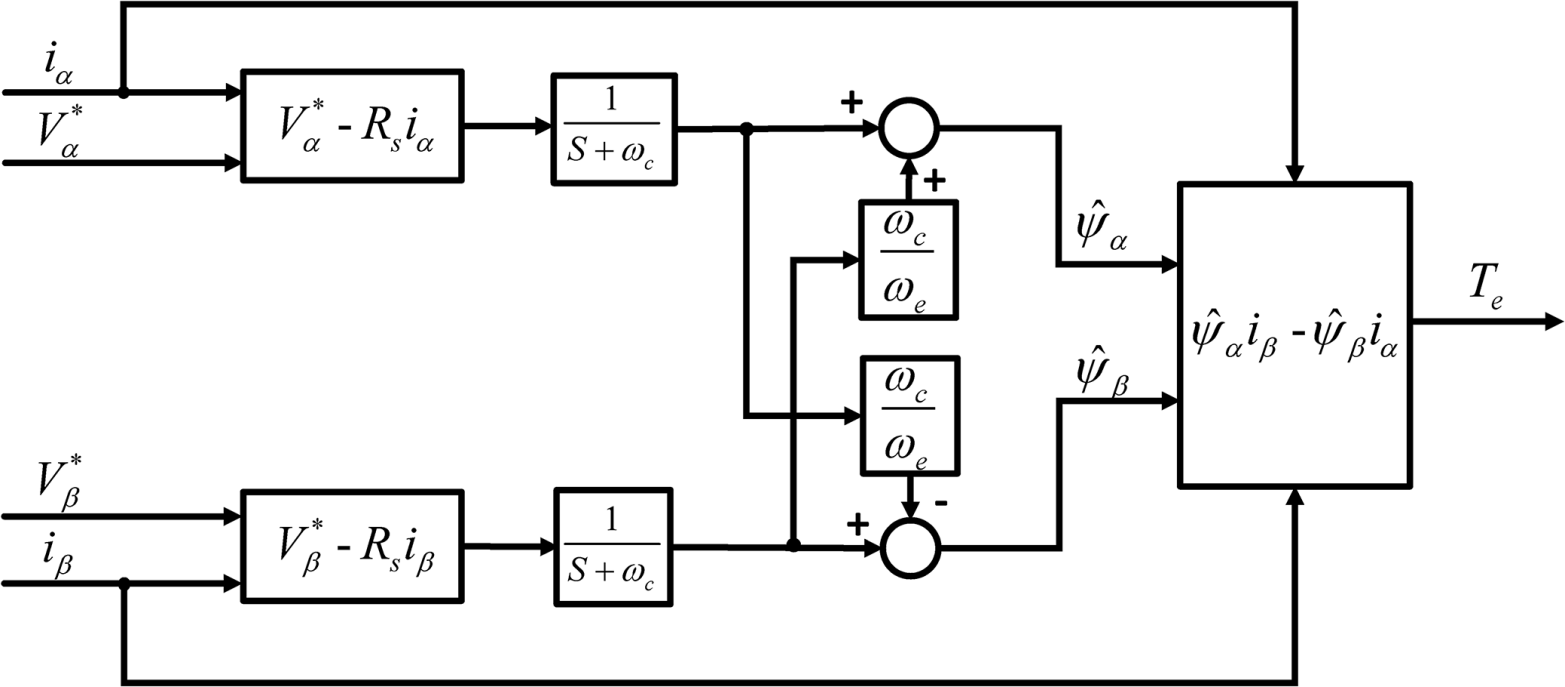
Flux Estimator with Compensated MLPF.

And according to Eq 3, Eq 7 and Eq 10, the schematic diagram of the torque estimator based on the compensated MLPF is shown in Fig 2.

In order to adapt different operation frequencies, variant cut off frequency *ω*_*c*_ can be implemented in MLPF, and it changes proportionally to the IPMSM operation frequency.

### Proposed Torque Estimation Method

#### Reference Voltage Correction

Because of the dead-time effect and the non-ideal characteristic of the inverter, reference voltages and actual stator voltages are not the same, and the voltage errors are relatively large when the motor is operating in low speed range. For better estimation accuracy, reference voltages have to be corrected before they can be used in the flux observer.

In [17, 18], models of the inverter are given. Actual mean phase to center voltages *V*_*as*_, *V*_*bs*_, *V*_*cs*_, can be expressed as:

$$\left\{ \begin{array}{l} V_{as}=V_{as}^{*}+V_{as}^{\prime }-\frac{1}{2}(r_{ce}+r_{d})i_{as}\\ V_{bs}=V_{bs}^{*}+V_{bs}^{\prime }-\frac{1}{2}(r_{ce}+r_{d})i_{bs}\\ V_{cs}=V_{cs}^{*}+V_{cs}^{\prime }-\frac{1}{2}(r_{ce}+r_{d})i_{cs} \end{array}\right.$$ (11)

where:

$$\left\{ \begin{array}{l} V_{as}^{\prime }=V\prime \{2sign(i_{as})-sign(i_{bs})-sign(i_{cs})\}\\ V_{bs}^{\prime }=V\prime \{2sign(i_{bs})-sign(i_{as})-sign(i_{cs})\}\\ V_{cs}^{\prime }=V\prime \{2sign(i_{cs})-sign(i_{as})-sign(i_{bs})\} \end{array}\right.$$ (12)

$$V\prime =\frac{1}{6}\left\{\frac{\left(V_{dc}-V_{ce}-V_{d})(T_{off}-T_{on}-T_{d}\right)}{T_{s}}-V_{ce}-V_{d}\right\}$$ (13)

$$sign(i_{as})=\left[\begin{array}{cc} 1:when & i_{as}>0\\ -1:when & i_{as}<0 \end{array}\right]$$ (14)

where *i*_*as*_, *i*_*bs*_, *i*_*cs*_ are phase currents respectively, and *V*_*ce*_ is threshold voltage of the active switch, *r*_*ce*_ is the on-state slope resistance of the active switch, *V*_*d*_ is threshold voltage of the freewheeling diode, *r*_*d*_ is the on-state slope resistance of the freewheeling diode, *T*_*s*_ is the sampling period, *T*_*on*_, *T*_*off*_ are turn on and turn off time of the power device, *T*_*d*_ is the dead-time of the PWM signal.

And *V*′ is the error voltage caused by the inverter, it can be calculated according to Eq 13.

According to the Clark transformation:

$$\left\{ \begin{array}{l} V_{\alpha }=V_{as}\\ V_{\beta }=\frac{\sqrt{3}}{3}V_{bs}-\frac{\sqrt{3}}{3}V_{cs} \end{array}\right.$$ (15)

$$\left\{ \begin{array}{l} V_{\alpha }^{*}=V_{as}^{*}\\ V_{\beta }^{*}=\frac{\sqrt{3}}{3}V_{bs}^{*}-\frac{\sqrt{3}}{3}V_{cs}^{*} \end{array}\right.$$ (16)

$$\left\{ \begin{array}{l} i_{\alpha }=i_{as}\\ i_{\beta }=\frac{\sqrt{3}}{3}i_{bs}-\frac{\sqrt{3}}{3}i_{cs} \end{array}\right.$$ (17)

The voltage correction method can be derived by substituting Eq 12–14 and Eq 16, Eq 17 into Eq 15:

$$\left\{ \begin{array}{l} V_{\alpha }=V_{\alpha }^{*}-\frac{1}{2}(r_{ce}+r_{d})\cdot i_{\alpha }+V\prime \{2sign(i_{as})-sign(i_{bs})-sign(i_{cs})\}\\ V_{\beta }=V_{\beta }^{*}-\frac{1}{2}(r_{ce}+r_{d})\cdot i_{\beta }+\sqrt{3}V\prime \{sign(i_{bs})-sign(i_{cs})\} \end{array}\right.$$ (18)

Eq 18 shows the relationship between actual stator voltages and reference voltages.

#### Torque Estimation Method with Voltage Correction

**Fig 3:**
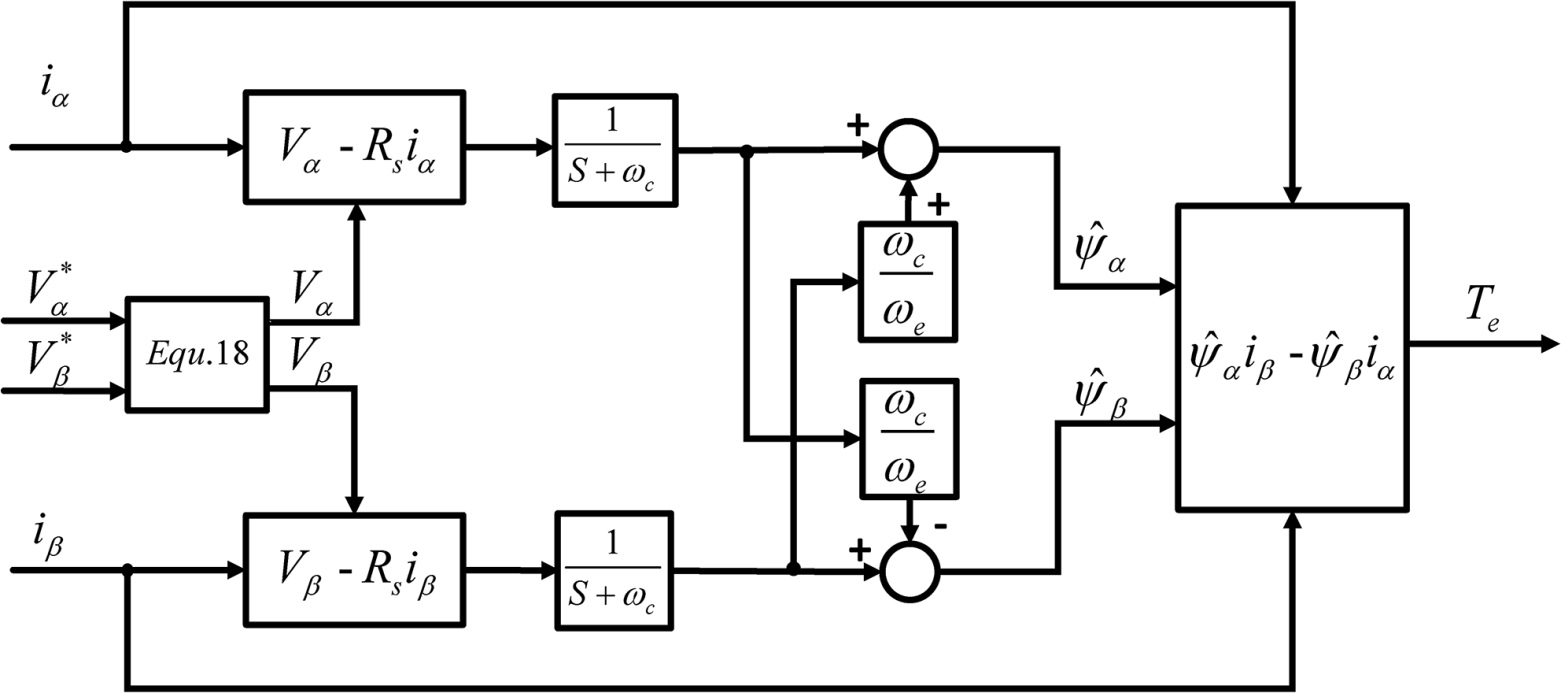
Proposed Torque Estimator with Reference Voltage Correction and Compensated MLPF.

Voltage errors between reference voltages and actual voltages will lead to torque estimation error. So if reference voltages are used in the torque estimation, voltage errors should be corrected. The proposed torque estimation with MLPF and reference voltage correction is shown in Fig 3.

In the proposed method, reference voltages of the inverter are corrected first according to Eq 18 to eliminate the error caused by the inverter. Then, MLPF is used for the flux estimation, and the cut off frequency of MLPF changes proportionally to the operation frequency of IPMSM. The output of MLPF is compensated according to Eq 10. In the end, the torque output of IPMSM will be calculated according to Eq 5.

## Results and Discussion

### Composition of the Verification System

**Fig 4:**
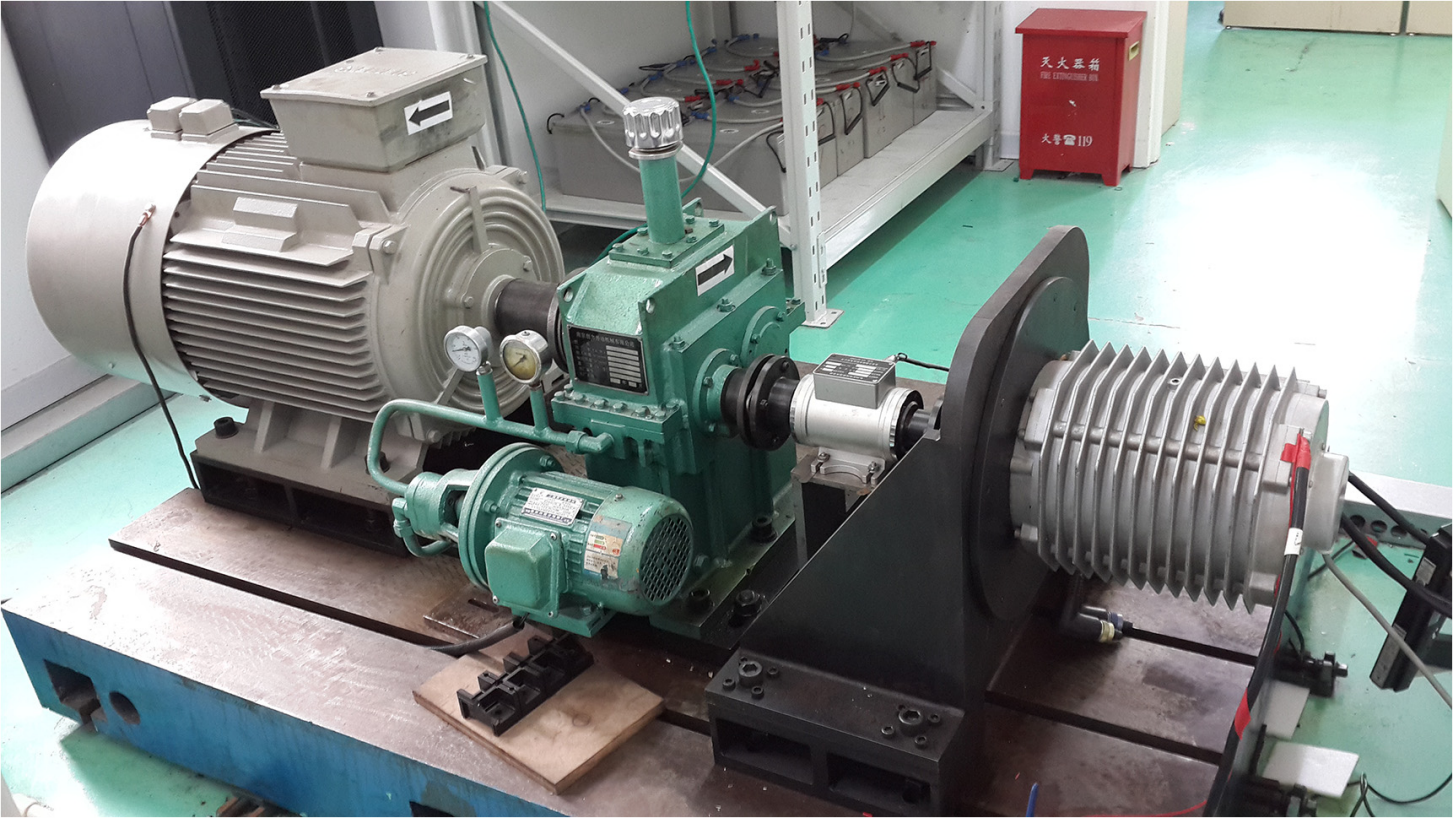
Experimental Setup.

In this section, the performance of the proposed method is verified by simulation and experimental results. Experiments are performed with a 47 kW PWM inverter fed IPMSM drive. The IPMSM is driven in the torque mode. An induction motor is used to apply the load torque to IPMSM, and is driven in the speed mode. The output torque of IPMSM can be read out from a torque sensor installed on the shaft. The measuring range of the torque senor is 200Nm with a measuring accuracy of 0.4Nm. The experimental setup is shown in Fig 4.

Simulation studies are carried out on MATLAB/Simulink platform. In the simulation, the IPMSM is driven in the current mode with a constant speed load, to simulate the mechanical load applied by the induction motor. Same parameters are used in the simulation and in the experiment, except the dead-time of the PWM signal. In order to demonstrate the influence of the inverter, the dead-time of the PWM signal in the simulation is larger than that in the experiment.

Parameters of the motor are provided in Table 1, and parameters of the inverter are provided in Table 2.

**Table 1:** Specifications of IPMSM.

**Pole pairs** *p*	4
**Stator resistance** *R*_*s*_	0.019[Ω]
**Flux linkage** *ψ*_*f*_	0.0865[Wb]
**Inductance of d axis** *L*_*d*_	0.381e-3[H]
**Inductance of q axis** *L*_*q*_	1.054e-3[H]
**Base speed** *n*_*b*_	4000[rpm]

**Table 2:** Specifications of Inverter.

**DC bus voltage** *V*_*dc*_	300[V]
**PWM frequency** *f*_*pwm*_	1e4[Hz]
**Dead-time** *T*_*d*_	5e-6[s] in simulation, 2e-6[s] in experiment
**Turn on time** *T*_*on*_	5.8e-7[s]
**Turn off time** *T*_*off*_	8.4e-7[s]
**on-state slope resistance of IGBT** *r*_*ce*_	2e-3[Ω]
**on-state slope resistance of diode** *r*_*d*_	2e-3[Ω]
**Saturation voltage of IGBT** *V*_*ce*_	0.9[V]
**Forward Voltage of diode** *V*_*d*_	0.9[V]

### Simulation Results

Simulation studies are carried out to verify the estimation accuracy of the proposed method. Results of the following three estimation methods are presented:

1. Torque estimator with MLPF and reference voltage correction, herein after referred as the proposed method.
2. Torque estimator with MLPF and without reference voltage correction, referred as the second method.
3. Torque estimator with a pure integrator and without reference voltage correction, referred as the third method.

In the third method, in order to reduce the influence of the initial value error, correct initial values are given to the integrator at the beginning of the simulation.

**Fig 5:**
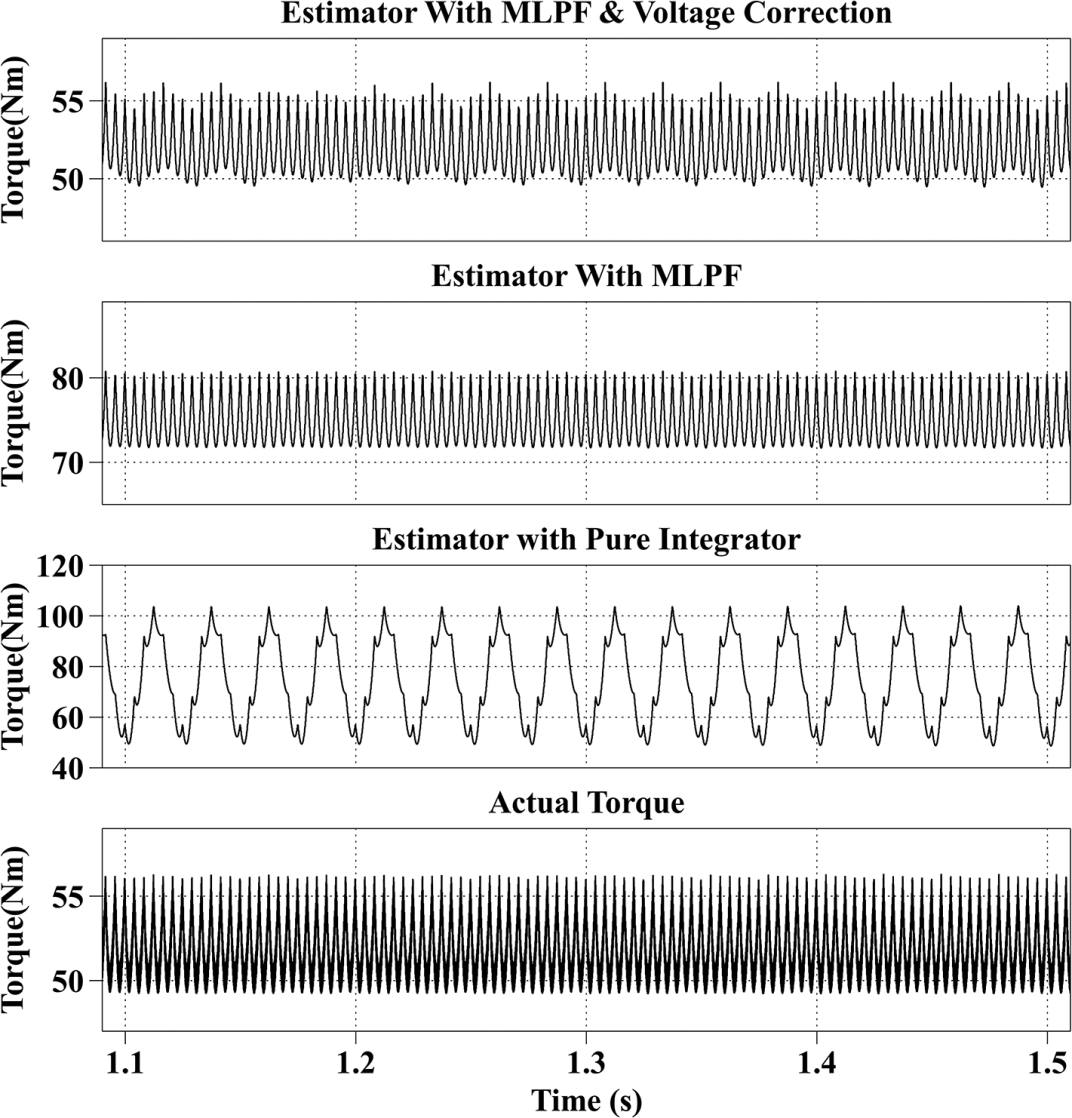
Torque Estimation Results with the Reference Current $i_{q}^{*}$ = 100A and the Reference Current $i_{d}^{*}$ = 0A at 600rpm.

Fig 5 shows the torque estimation results of the three different methods at 600rpm with the reference current $i_{q}^{*}$ of 100A and the reference current $i_{d}^{*}$ of 0A. The actual torque output is from 49Nm to 57Nm, and the estimated torque with the proposed method is from 50Nm to 57Nm, with an average estimation error within 1Nm. Because current signals are sampled discretely every 100μs, so the torque ripple caused by the current ripple cannot be reconstructed. The average estimation error of the second method is 23Nm, and is obviously larger than the actual torque output, because voltage errors caused by the dead-time effect account for a large proportion of the reference voltages when the motor is operating in the low speed range. And the result of the third method is not stable, the estimated torque fluctuates from 50Nm to 110Nm due to the DC drift, although a correct initial value is given to the integrator.

**Fig 6:**
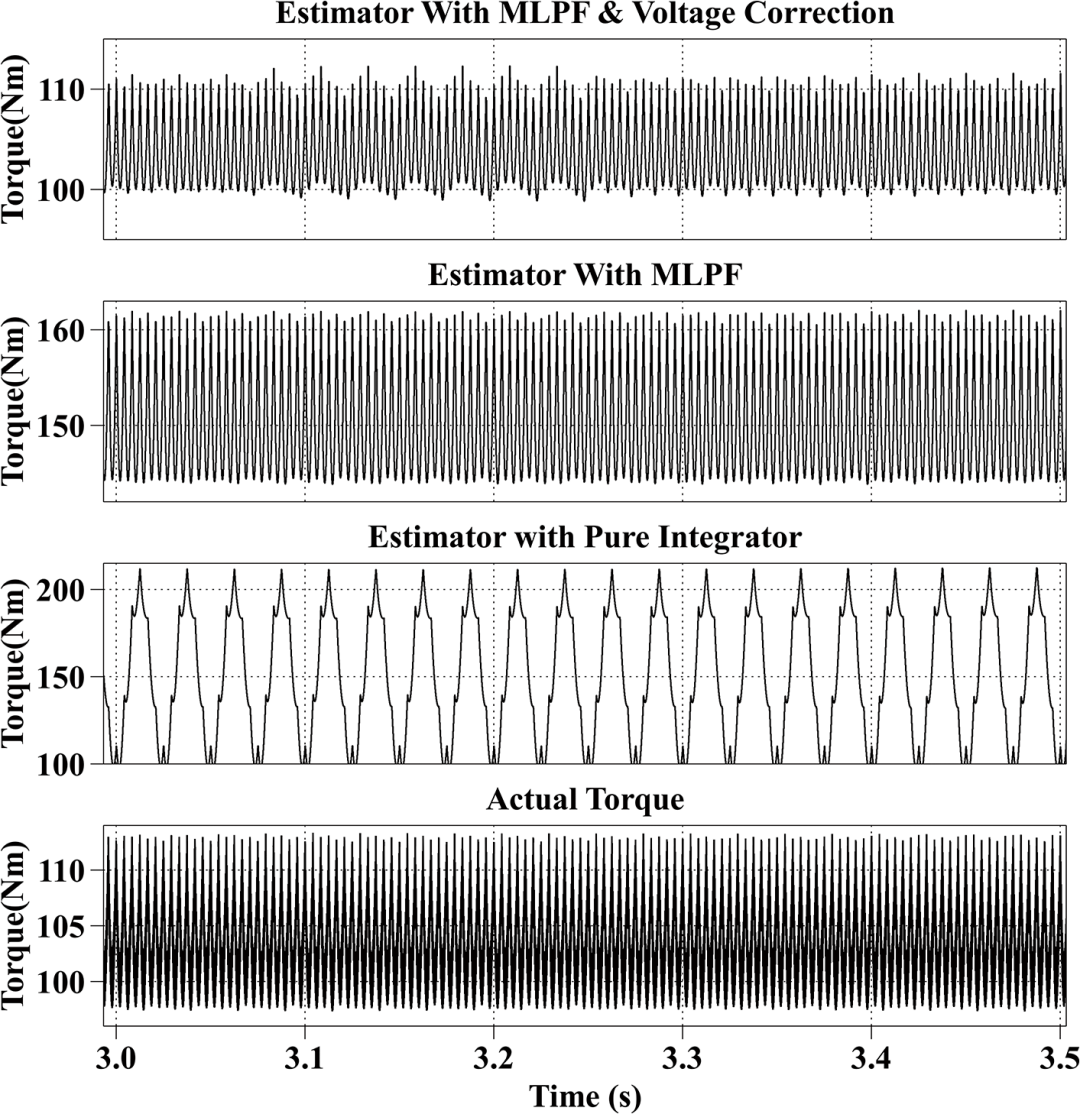
Torque Estimation Results with the Reference Current $i_{q}^{*}$ = 200A and the Reference Current $i_{d}^{*}$ = 0A at 600rpm.

Fig 6 shows the estimation results at 600rpm with the reference current $i_{q}^{*}$ of 200A and the reference current $i_{d}^{*}$ of 0A. The actual torque output is from 98Nm to 113Nm. The average estimation error of the proposed method is within 1Nm. The estimation result of the second method is much larger than the actual torque output, with an average error of 47Nm, which shows the necessity of the voltage correction. The estimated result of the third method fluctuates from 100Nm to 200Nm.

**Fig 7:**
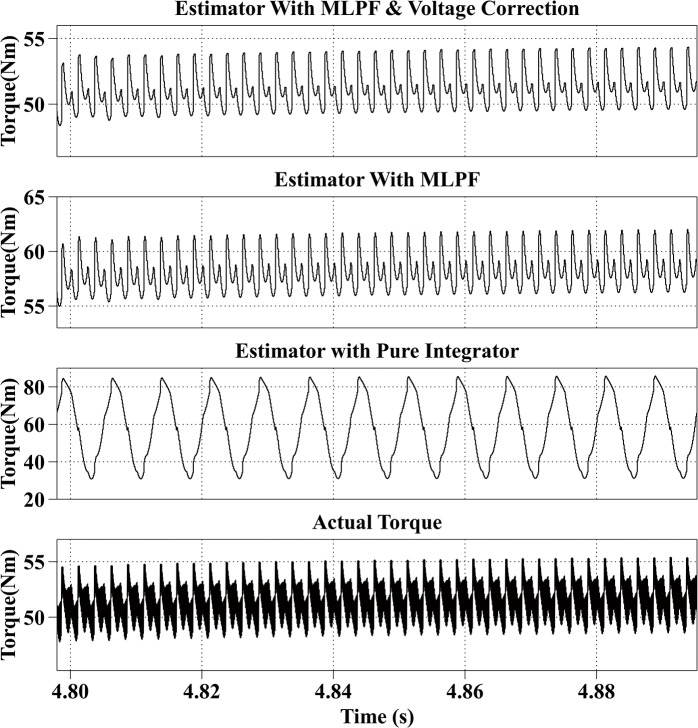
Torque Estimation Results with the Reference Current $i_{q}^{*}$ = 100A and the Reference Current $i_{d}^{*}$ = 0A at 2000rpm.

Fig 7 shows the estimation results at 2000rpm with the reference current $i_{q}^{*}$ of 100A and the reference current $i_{d}^{*}$ of 0A. The actual torque output is from 48Nm to 55Nm. The average estimation error of the proposed method is within 1Nm. The average error of the second method is 6Nm and is smaller than the error when the motor is operating in the low speed range, because the BEMF increases with the speed and makes voltage errors relatively smaller. The estimated result of the third method fluctuates from 100Nm to 200Nm.

**Fig 8:**
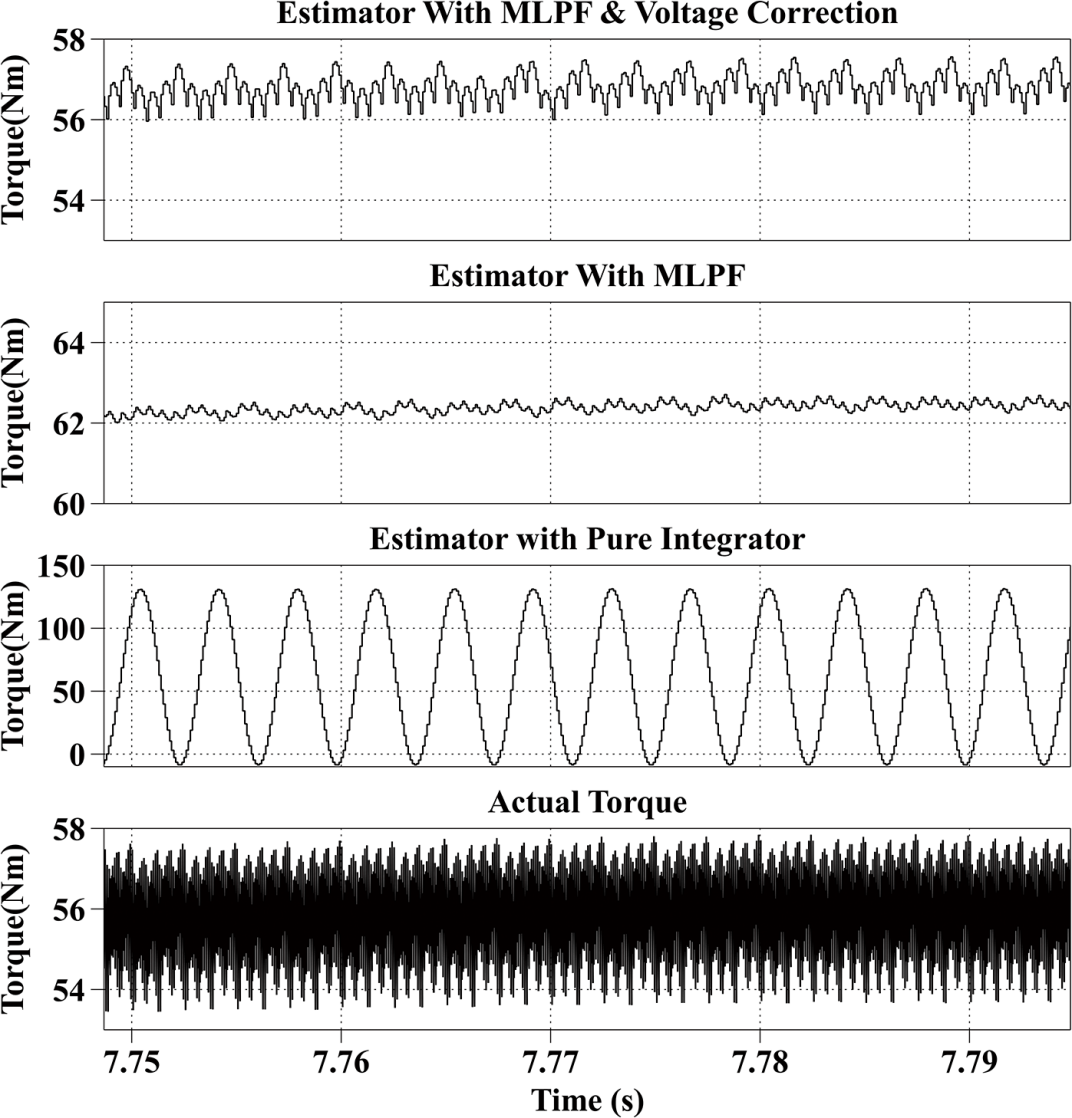
Torque Estimation Results with the Reference Current $i_{q}^{*}$ = 50A and the Reference Current $i_{d}^{*}$ = -150A at 4000rpm.

Fig 8 shows the estimation results at 4000rpm with the reference current $i_{q}^{*}$ of 50A and the reference current $i_{d}^{*}$ of -150A, the PM motor is operating in flux weakening region. The actual torque output is from 53Nm to 58Nm. The average estimation error of the proposed method is within 2Nm, and the average estimation error of the second method is 7Nm. The estimated result of the third method fluctuates from 0Nm to 130Nm.

All simulation results are summarized in Table 3. From the simulation results, it can be observed that the proposed method has the best estimation accuracy. And because of the dead-time effect, the estimated torque with the second method is bigger than the actual torque. And the result of the third method is not stable.

**Table 3:** Simulation Results.

Speed (rpm)	Reference currents (A)	Actual torque （Nm）	Estimated torque with proposed method (Nm)	Estimated torque with MLPF (Nm)	Estimated torque with pure integrator (Nm)
600	$i_{q}^{*}$ = 100, $i_{d}^{*}$ = 0	49~57	50~57	72~80	50~100
600	$i_{q}^{*}$ = 200, $i_{d}^{*}$ = 0	98~113	100~110	145~161	100~200
2000	$i_{q}^{*}$ = 100, $i_{d}^{*}$ = 0	48~55	48~55	55~63	30~84
4000	$i_{q}^{*}$ = 50, $i_{d}^{*}$ = -150	53~58	56~57	62~63	0~130

### Experimental Results

Same estimation methods are implemented and compared in the experiment.

**Fig 9:**
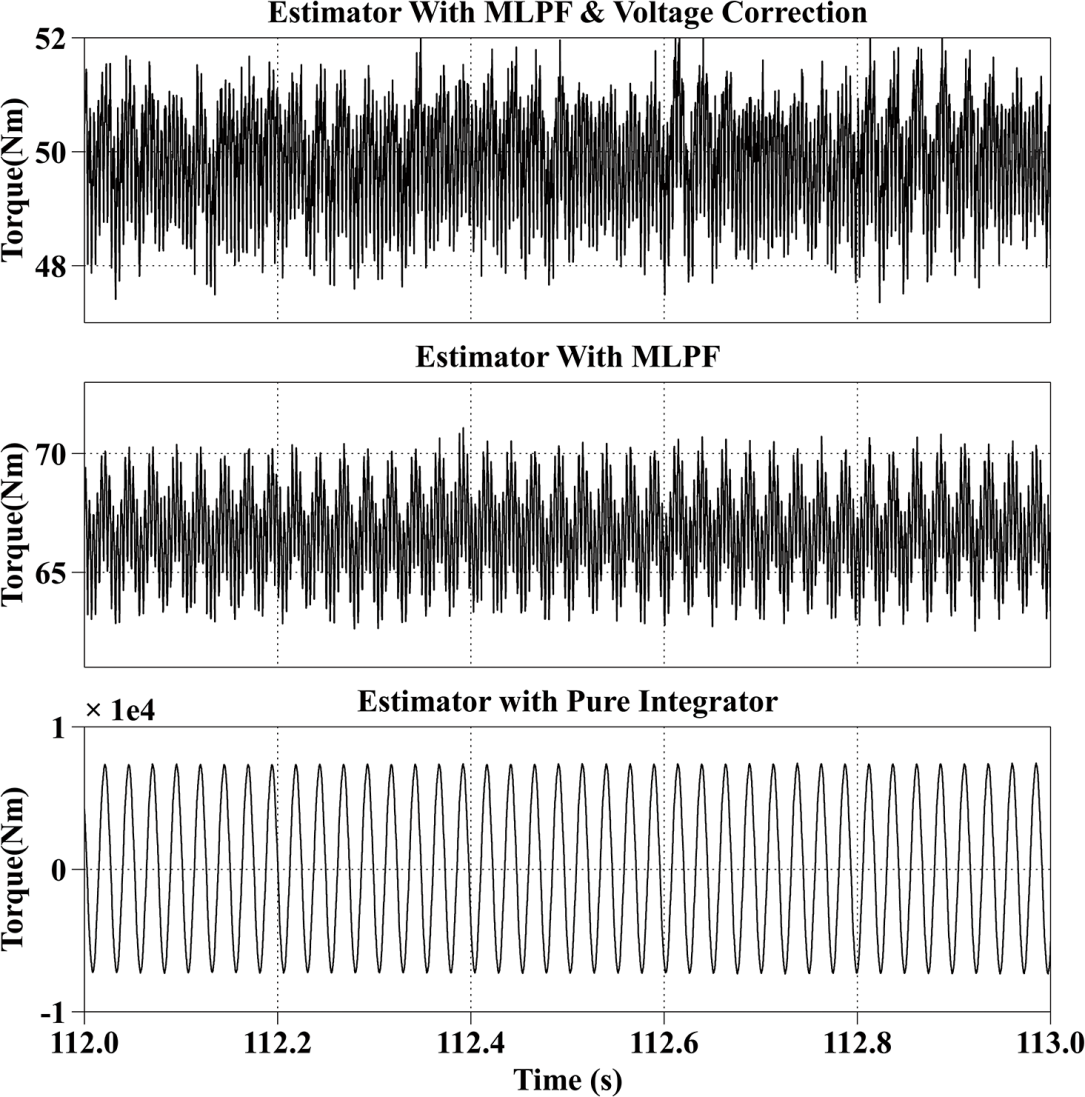
Torque Estimation Results at 600rpm with a Measured Torque of 50Nm.

Fig 9 shows the torque estimation results of the three methods at 600rpm with a measured torque of 50Nm from the torque sensor. And the average estimation error of the proposed method is 1Nm. The average estimation error of the second method is 17Nm. And the result of the third method is not stable, because the experiment takes longer time than the simulation and the error of the integrator accumulates to a very big value.

**Fig 10:**
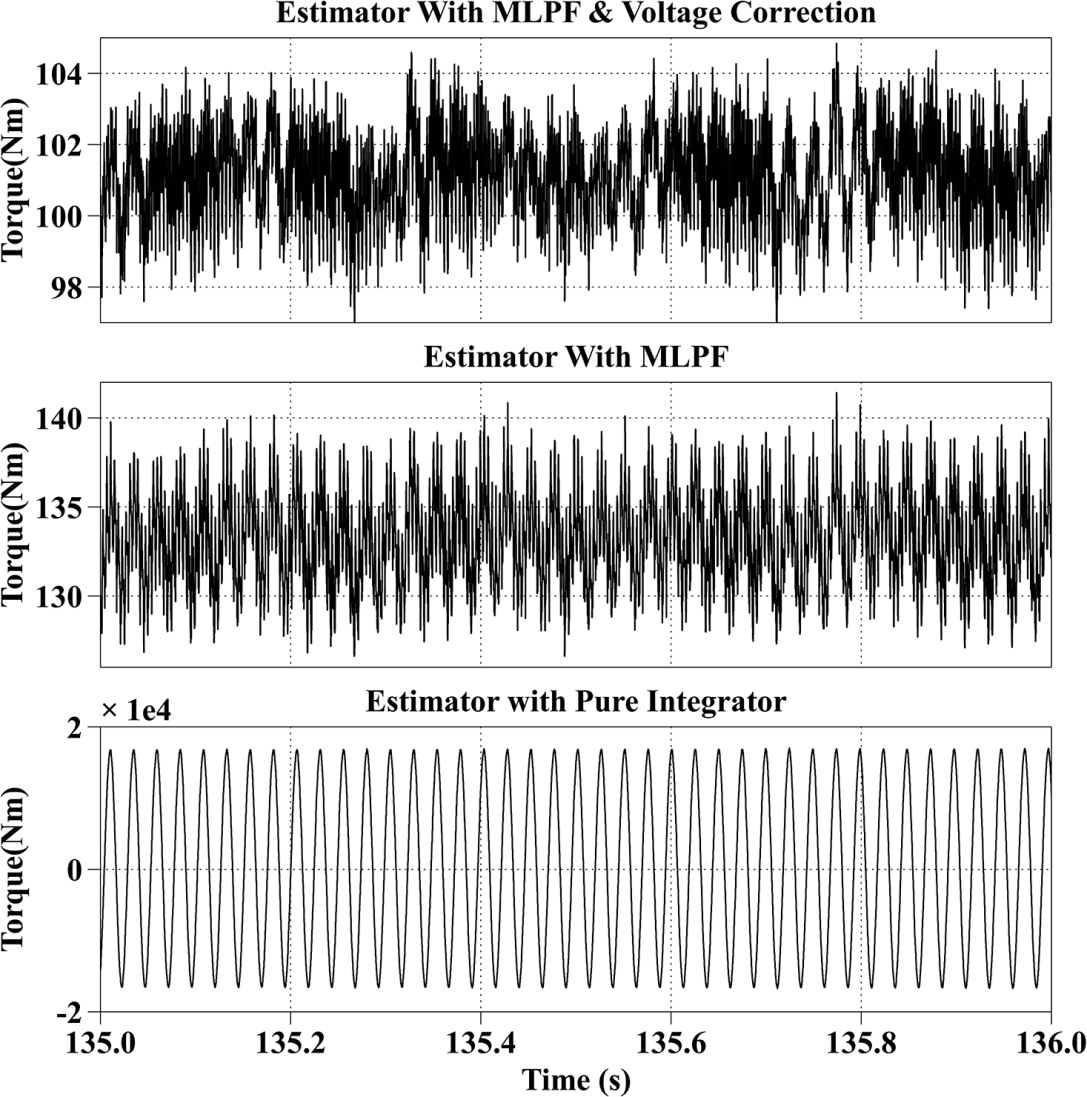
Torque Estimation Results at 600rpm with a Measured Torque of 100Nm.

Fig 10 shows the torque estimation results at 600rpm with a measured torque of 100Nm. And the average errors of the proposed method and the second method are 1Nm and 34Nm respectively. And the result of the third method is not stable.

**Fig 11:**
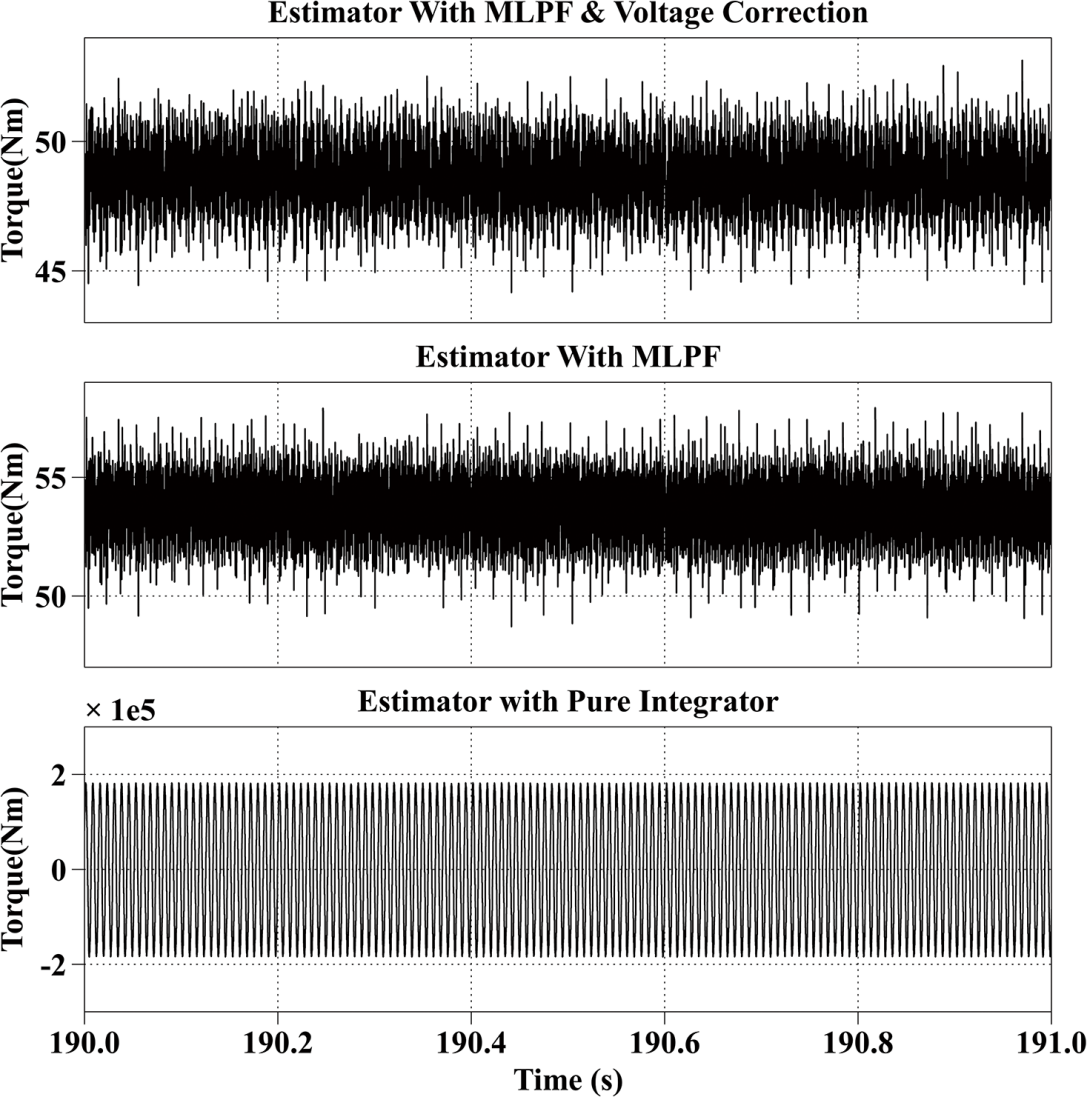
Torque Estimation Results at 2000rpm with a Measured Torque of 50Nm.

Fig 11 shows the torque estimation results at 2000rpm with a measured torque of 50Nm. And the average errors of the first two methods are 1Nm and 4Nm. And the result of the third method is not stable.

**Fig 12:**
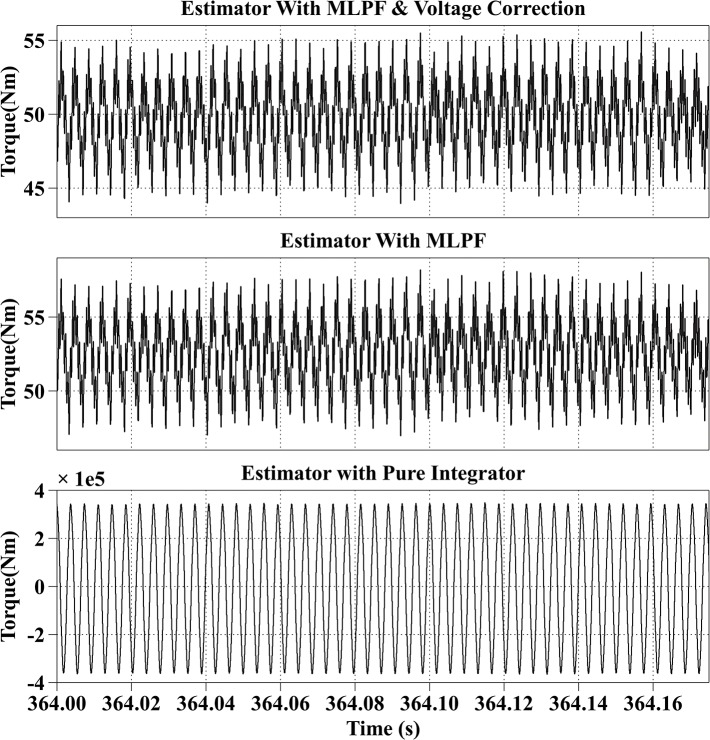
Torque Estimation Results at 4000rpm with a Measured Torque of 50Nm.

Fig 12 shows the torque estimation results at 4000rpm with a measured torque of 50Nm. The average error of the proposed method is within 1Nm and the second method is within 3Nm. And the result of the third method is not stable.

All experimental results are summarized in Table 4. From the experimental results, it can be seen that the accuracy of the proposed method is better than the other two methods. And the experimental results of the second method are better than simulation results, because the dead-time is smaller, it also implies that the estimation results are influenced by the dead-time of the inverter. The experimental results of the third method are even worse than simulation results, because the experiments take longer time than simulations, and the error of the integrator grows with the time.

**Table 4:** Experimental Results.

Speed (rpm)	Measured torque from torque sensor (Nm)	Estimated torque with proposed method (Nm)	Estimated torque with MLPF (Nm)	Estimated torque with pure integrator (Nm)
600	50	47~52	63~70	-0.8e4 ~ 0.8e4
600	100	98~104	133~140	-1.8e4 ~ 1.8e4
2000	50	45~53	50~57	-1.9e5 ~ 1.9e5
4000	50	44~55	47~58	-3.8e5 ~ 3.8e5

## Conclusions

This paper proposes a torque estimation method of IPMSM based on the stator flux observer. With MLPF, the influence of the initial error and the DC drift is eliminated. The phase error and the gain error of MLPF are also compensated.

Moreover, errors between reference voltages and actual stator voltages caused by the dead-time effect and the non-ideal characteristic of the inverter are analyzed and corrected. Compared with methods without considering voltage errors, the proposed method significantly improves the estimation accuracy, especially when the motor is operating in the low speed range and voltage errors account for a big proportion of reference voltages.

The proposed method is simple and practical, and no extra hardware modification is needed. It has been simulated on MATLAB/Simulink platform and implemented on actual 47 kW IPMSM drive system. Simulation and experimental results show considerable improvements in the estimation accuracy, thus verifying the effectiveness of the proposed method.

## Supporting Information

S1 Appendix List of symbols (nomenclature). (DOCX)
