# Supplementary material for: A Practical Torque Estimation Method for Interior Permanent Magnet Synchronous Machine in Electric Vehicles
Source: PLoS One. 2015 Jun 26;10(6):e0130923. doi: 10.1371/journal.pone.0130923 (PMC4482678; doi:10.1371/journal.pone.0130923)
Supplement: S1 Appendix — (DOCX) [file pone.0130923.s001.docx]

**List of symbols (nomenclature)**

$V_{\alpha}$, $V_{\beta}$ Actual $\alpha$ $\beta$-axis voltages (in volts).

$R_{s}$ Stator winding resistance (in ohms).

$i_{\alpha}$, $i_{\beta}$ Actual $\alpha$ $\beta$-axis currents (in amperes).

$\psi_{\alpha}$, $\psi_{\beta}$ Actual $\alpha$ $\beta$-axis flux linkages (in webers).

$\hat{\psi}_{\alpha}$, $\hat{\psi}_{\beta}$ Estimated $\alpha$ $\beta$-axis flux linkages (in webers).

$\hat{T}_{e}$ Estimated electric torque (in newton meters).

$p$ Number of pole pairs.

$\omega_{c}$ Cutoff frequency of modified low pass filter (in radians per second).

$\omega_{e}$ Synchronous speed (in radians per second).

Phase lag of the MLPF without compensation (in degrees).

$M$ Gain $M$ of the MLPF without compensation.

$\hat{\psi}'_{\alpha}$,$\hat{\psi}'_{\beta}$ Estimated $\alpha$ $\beta$-axis flux linkages of the MLPF without compensation (in webers)

$V_{as}$,$V_{bs}$,$V_{cs}$ Actual abc phase line-to-neutral voltages (in volts).

$V_{as}^{*}$,$V_{bs}^{*}$,$V_{cs}^{*}$ Abc phase reference voltages (in volts).

$V'_{as}$,$V'_{bs}$,$V'_{cs}$ Distorted abc phase voltages (in volts).

$V'$ Error voltage caused by inverter (in volts).

$r_{ce}$ On-state slope resistance of the active switch (in ohms).

$r_{d}$ On-state slope resistance of the freewheeling diode (in ohms).

$i_{as}$,$i_{bs}$,$i_{cs}$ Actual abc phase currents (in amperes).

$V_{dc}$ DC bus voltage (in volts).

$V_{ce}$ Threshold voltage of the active switch (in volts).

$V_{d}$ Threshold voltage of the freewheeling diode (in volts).

$T_{off}$,$T_{on}$ Turn-on/turn-off times of insulated-gate bipolar transistor (IGBT) (in seconds).

$T_{d}$ Dead time of the switch (in seconds).

$T_{s}$ Sampling period (in seconds).

$V_{\alpha}^{*}$, -axis reference voltages (in volts).

$\psi_{f}$ Rotor flux linkage (in webers).

$L_{d}$,$L_{q}$ dq-axis inductances (in henrys).

$n_{b}$ Base speed (in rounds per second).

$f_{pwm}$ PWM frequency (in hertzs).

$i_{d}^{*}$,$i_{q}^{*}$ dq-axis reference currents (in amperes).
